# Child maltreatment across the lifespan

**DOI:** 10.1080/20008198.2018.1483165

**Published:** 2018-08-02

**Authors:** Maj Hansen, Miranda Olff

**Affiliations:** a ThRIVE, Department of Psychology, University of Southern Denmark, Odense, Denmark; b Department of Psychiatry, Academic Medical Center at the University of Amsterdam, Amsterdam, The Netherlands; c Arq Psychotrauma Expert Group, Diemen, The Netherlands

**Keywords:** Child maltreatment, ESTSS 2017, globalization, diagnostics, PTSD, ICD-11, • The present editorial highlights research presented at the Annual Meeting of ESTSS 2017, ‘Child Maltreatment across the Lifespan’.• It underlines important steps taken towards a biological and systemic understanding, global collaboration, and a more uniform clinical approach to child maltreatment.• An update on ICD-11 and implications of implementing ICD-11 are included.

## Abstract

The present editorial introduces selected research for a special issue covering the 15th Annual Meeting of the European Society for Traumatic Stress Studies (ESTSS) 2017, ‘Child Maltreatment across the Lifespan’. Unfortunately, childhood maltreatment is highly prevalent around the world. Exposure to child maltreatment is associated with a considerable risk of developing a wide range of psychopathologies. Thus, preventing child maltreatment and offering early treatment to victims is an important and promising area in reducing the risk of psychopathology. The 10 articles included in the special issue focus on different important areas related to the topic of child maltreatment. These include a biological and systemic understanding of maltreatment, a globalization perspective, and contributions on diagnosis and treatment, with implications for clinical practice. Furthermore, the special issue includes an update on ICD-11 and implications associated with the implementation of ICD-11. Future research recommendations are provided to underline the enduring need to promote and undertake research into all aspects of child maltreatment.

## 1. Introduction

The main theme of the 15th Annual Meeting of the European Society for Traumatic Stress Studies (ESTSS) 2017 was ‘Child Maltreatment across the Lifespan’. Thus, the primary goal of the conference was to establish current knowledge and identify future directions for research on child maltreatment. The conference was held at the University of Southern Denmark in Odense, on 2–4 June. Odense was the hometown of the famous author and poet Hans Christian Andersen, who touched on the conference theme in several aspects of his works, including the fairy tale of the ugly duckling:

*“So it went on the first day, and after that things get worse and worse. The poor duckling was chased by everyone. Even his own siblings were mean to him. They would always say, ‘how we wish the cat would catch you, you nasty thing.’ And his mother said, ‘How I do wish you were miles away. And the ducks bit him, the hen picked him, and the girl, feeding the animals, kicked him.* (Translated from Andersen, 2014, pp. 15–16).

The fairy tale of the ugly duckling ends happily with the ugly duckling realizing that it is a beautiful swan and finding its own kind. Unfortunately, childhood maltreatment does not always end happily in real life.

Child maltreatment can generally be defined as all types of abuse, i.e. sexual, physical, and emotional, and neglect of a child (Elklit, Karstoft, Armour, Feddern, & Christofferson, 2013). Unfortunately, childhood maltreatment is generally highly prevalent across the world (Stoltenborgh, Bakermans-Kranenburg, Alink, & van Ijzendoorn, 2015). Exposure to child maltreatment is associated with a considerable risk of developing post-traumatic stress disorder (PTSD) and complex PTSD (CPTSD) as well as a wide range of other forms of psychopathology (Ford, 2015). Thus, preventing child maltreatment and offering early treatment to victims are important and promising areas in reducing the risk of psychopathology.

The present special issue includes 10 invited papers that are based on various presentations at the ESTSS 2017 relating in different ways to the main conference theme of child maltreatment.

## 2. Childhood maltreatment

The editorial by Danese and van Harmelen (2017) briefly comments on how childhood trauma can be translated into a biological risk for psychopathology (i.e. hidden wounds), which may aid the understanding of childhood trauma. Specifically, childhood trauma may have a long-term impact on the immune system (see also Olff & van Zuiden, 2017) as a result of greater amygdala reactivity to emotional stimuli, which activates immune cells and the enduring systemic inflammatory response. Danese and van Harmelen (2017) describe the associated clinical implications in terms of both secondary and tertiary prevention if inflammation explains the link between childhood trauma and later mental and physical health.

Teicher et al. (2018) investigated whether abnormalities in sleep continuity have effects on brain morphometry and whether sleep impairments mediate the effects of child maltreatment on the brain structure in the late teens. Child maltreatment was found to be significantly associated with several different indicators of disruptive effects on sleep, and exposure to parental non-verbal emotional abuse was the most important predictor of impaired sleep. Furthermore, reduced sleep efficiency was associated with several changes in brain morphometry, and sleep was found to mediate 39–46% of the effect of maltreatment on the volume of hippocampal structures and the inferior frontal cortex. These findings suggest that inventions targeting sleep enhancement may reduce the negative neurobiological effects of maltreatment.

Moving away from biological areas of child maltreatment, Punimäki, Qouta, and Peltonen (2018) underline the need to explore the effect of trauma from a transgenerational family perspective. Punamäki et al. (2018) identified four different family types associated with different attachment patterns, sibling relations, and parental practices in Palestinian families exposed to the war in Gaza. In general, family relationships characterized by insecure attachment, sibling conflicts, and negative parental practices were associated with higher severity parental war trauma and poorer mental health in children. The clinical implications are discussed and point to a need for interventions to include a focus on improving family relations among war-affected children.

Similarly, the Young Minds Paper in a Day Workshop also focused on child maltreatment from a transgenerational perspective. Specifically, Christie et al. (2018) reviewed the literature on how child maltreatment may affect transition to parenthood. The scoping review by Christie et al. (2018) identified 69 studies, and showed that experiences of child maltreatment appear to have negative effects on several aspects of parental mental health, bodily and physical changes, and parental view of the child, but mixed results were found in relation to the view of self as a parent. The review concludes on the complexities surrounding the transition into parenthood for parents with a childhood trauma history and the associated clinical implications.

Finally, Deblinger and Pollio (2018) describe and review treatment research outcomes of trauma-focused cognitive behavioural therapy (TF-CBT) applied to young children and their caregivers. At the same time, Deblinger and Pollio (2018) discuss developmental and clinical considerations when applying TF-CBT in young populations. The authors underline that when implementing TF-CBT it is important to make considerations to enhance treatment outcome. These include shorter sessions, lasting 20–30 minutes, conjoint sessions with children and their caregivers, and the use of play and music. The paper concludes with a brief review of research supporting the effects of TF-CBT.

The paper by Schnyder et al. (2017) describes the establishment of a global collaboration of all major societies on traumatic stress studies, such as the ESTSS, the Argentina Society for Psychotrauma, the Asian Society for Traumatic Stress Studies, the Australasian Society for Traumatic Stress Studies, the Canadian Psychological Association Traumatic Stress Section, the German-speaking Society for Psychotraumatology, the International Society for Traumatic Stress Studies (ISTSS), and the Japanese Society for Traumatic Stress Studies. The initiative was launched by the ISTSS. Schnyder et al. (2017) highlight two results of the collaboration so far: internet information on Childhood Abuse and Neglect (iCAN) and the computerized Childhood Attachment and Relational Trauma Screen (CARTS). The iCAN provides information for adult individuals affected by childhood abuse and neglect, whereas the CARTS is a self-report measure designed to measure occurrences of childhood maltreatment (Frewen et al., 2013, 2015). Both are available in several languages (see https://www.estss.org/public-education-epamphlets/or, e.g. www.estss.org/german-abstract/). As Schnyder et al. (2017) note, the collaboration can be seen as encouraging steps towards a more global structure in the field of traumatic stress. As trauma is a global issue (Hall & Olff, 2016; Kessler et al., 2017; Magruder, McLaughlin, & Borbon, 2017), global collaboration on important topics seems an efficient way to move the field forward.

The Horizon 2020, Marie Skłodowska-Curie Actions-funded, research and training programme called CONTEXT (COllaborative Network for Training and EXcellence in psychoTraumatology) can also be seen as a step towards the globalization of psychotraumatology (Vallières et al., 2018). CONTEXT is a collaboration between a consortium of three universities and six non-academic partners equally divided across three European counties (Denmark, Ireland, and the UK). Vallières et al. (2018) describe the three main objectives of CONTEXT: research, training, and implementation. The research consists of 12 interconnected doctoral research projects within three themes. These themes are Survivors and Perpetrators of Childhood and Gender-based Violence, Refugees and Asylum Seekers, and First Responders. The training consists of both formal training [e.g. 30 European Credit Transfer and Accumulation System (ECTS) points full credited programmes taught by the experts of the consortium]and informal training (e.g. working within different contexts of both academia and practice). Finally, Vallières et al. (2018) comment on the dissemination of CONTEXT, which is to be directed towards improving policy, guidelines, and practice within the field of psychotraumatology.

The 11th revision of the International Classification of Diseases (ICD-11) is soon to be released, and offers alternative descriptions of trauma-related disorders to the *Diagnostic and Statistical Manual of Mental Disorders*, 5th edition (DSM-5, APA, 2013; Hansen, Hyland, Armour, Shevlin, & Elklit, 2015). Specifically, in relation to PTSD, the Hansen et al. (2017) study aims at investigating the potential impact of choosing one rather than the other diagnostic system across three distinct trauma samples (*N* = 4213). Results showed that although the only significant difference in the estimated prevalence rates was found in the university sample (i.e. a higher DSM-5 PTSD rate compared to the ICD-11 PTSD rate), only moderate agreement was found between the two diagnostic systems (i.e. kappa = .60–.68). Furthermore, the three-factor ICD-11 model provided the best fit of the data from the tested models of the latent structure of ICD-11 PTSD symptoms, whereas two alternative models (i.e. the Hybrid model and the Anhedonia model) provided the best fit of the data from the tested models of the latent structure of DSM-5 PTSD symptoms. Hansen et al. (2017) discuss the implications of use of the two diagnostic systems, which is not straightforward as the estimated PTSD rates may be both qualitatively and quantitively influenced by this choice.

Kazlaukas, Zelviene, Lorenz, Quero, and Maercker (2017) provide a scoping literature review on studies of the proposed revised ICD-11 conceptualization of adjustment disorder (AjD), including a brief overview of the historical background, AjD measurements, and the treatment studies. The review identifies 10 studies, which are focused on measurement validation, the latent structure of AjD, predictors of AjD, and/or treatment of AjD. Thus, the review identified limited research in this area and the need for more research is highlighted and discussed. However, the review concludes that the updates to AjD definition in the ICD-11 could significantly enhance the understanding of AjD.

Finally, the paper by Karatzias al. (2017) provides an update of the conceptual structure and self-report measurement of PTSD and CPTSD using the International Trauma Questionnaire (ITQ), with a focus on selected studies from the USA, Germany, the UK, and Lithuania. Preliminary results suggest that CPTSD is common across the four countries in both clinical and general population samples, and that the ITQ can distinguish between PTSD and CPTSD (see also Hansen et al., 2017). Furthermore, the ITQ appears to be a reliable and valid measurement. However, more research is needed, especially in relation to generating the final version of the ITQ to ensure the matching of the diagnostic structure of PTSD and CPTSD across cultures.

## 3. Recommendations for future research

The studies included in this special issue covering the ESTSS 2017, alongside other research presented at the ESTSS 2017 and the current debates in research, politics, media, and so on, underline that the definition of child maltreatment varies both within and between cultures and borders. For instance, in some countries and cultures corporal punishment of a child is considered part of parenting and acceptable, while in other countries it is considered child maltreatment and illegal. Regardless of the surrounding culture, norms, and legislation, exposure to child maltreatment is associated with a wide range of psychological reactions, and affects not only the child but also the network around the child. This is complicated further by the fact that victims of child maltreatment may be revictimized and exposed to multiple traumatic events (i.e. polyvictimization), as well as being at increased risk of committing violence towards others. Furthermore, although emotional abuse is known to have damaging effects, such abuse is difficult to identify. This makes it especially challenging to assess, prevent, treat, and introduce legislation in this area, which is currently being debated in Denmark. The present issue underlines the need to see the full picture of the effects of child maltreatment and not focus solely on isolated features in a time-limited perspective. At the same time, there is a need for a uniform definition, assessment, diagnostics, and treatment of child maltreatment. Important steps have been taken in this area to improve global collaboration and to provide a more uniform approach to child maltreatment, but there is still a need to build on the established knowledge and expertise to promote further research and develop practice within this field.
